# The rise of *Candidozyma auris* in Czechia: three clades, prosthetic joint infection and fluconazole resistance development, 2022 to 2024

**DOI:** 10.2807/1560-7917.ES.2025.30.45.2500285

**Published:** 2025-11-13

**Authors:** Bram Spruijtenburg, Jacques F Meis, Norman van Rhijn, Martina Čurdová, Eva Kašperová, Petr Vašek, Lucie Bartoníčková, Jan Kubele, Petra Olišarová, Kateřina Svobodová, Daniela Lžičařová, Dana Němcová, Věra Kůrková, Šárka Lásiková, Naďa Mallátová, Theun de Groot, Pavlína Lysková, Eelco F J Meijer

**Affiliations:** 1 Radboudumc-CWZ Center of Expertise for Mycology, Nijmegen, The Netherlands; 2 Department of Medical Microbiology and Immunology, Canisius-Wilhelmina Hospital (CWZ)/Dicoon, Nijmegen, The Netherlands; 3 Institute of Translational Research, Cologne Excellence Cluster on Cellular Stress Responses in Aging-Associated Diseases (CECAD) and Excellence Center for Medical Mycology, University of Cologne, Cologne, Germany; 4 Manchester Fungal Infection Group, Division of Evolution, Infection and Genomics, Faculty of Biology, Medicine and Health, University of Manchester, Manchester, United Kingdom; 5 Microbial Evolution Research Manchester, Division of Evolution, Infection and Genomics, Faculty of Biology, Medicine and Health, University of Manchester, Manchester, United Kingdom; 6 Department of Microbiology, Military University Hospital, Prague, Czechia; 7 Department of Orthopedics, First Faculty of Medicine, Charles University and Military Hospital Prague, Czechia; 8 Department of Medical Microbiology, University Hospital Královské Vinohrady, Prague, Czechia; 9 Department of Medical Microbiology, Homolka Hospital, Prague, Czechia; 10 Department of Clinical Microbiology and ATB Centre, Institute of Medical Biochemistry and Laboratory Diagnostics, General University Hospital in Prague, Prague, Czechia; 11 Department of Medical Microbiology, 2nd Faculty of Medicine, Charles University in Prague and Motol University Hospital, Prague, Czechia; 12 Department of Medical Microbiology, Písek Hospital, Písek, Czechia; 13 Department of Medical Microbiology, Bulovka Hospital, Prague, Czechia; 14 Department of Medical Microbiology, České Budějovice Hospital, České Budějovice, Czechia; 15 Department of Medical Microbiology Prague and Kladno, Public Health Institute in Ústí nad Labem, Prague, Czechia

**Keywords:** *Candida auris*, *Candidozyma auris*, antifungal resistance, outbreak, genotyping, whole genome sequencing, fluconazole

## Abstract

**BACKGROUND:**

*Candidozyma auris* has emerged globally as a major threat to public health due to its outbreak causing capacity and antifungal resistance. Outbreaks have proven difficult to control despite enhanced infection prevention measures. Thus, national surveillance is warranted.

**AIM:**

We aim to characterise the epidemiology of *C. auris* cases in Czechia between 2022 and 2024 to investigate whether autochthonous spread is occurring and asses antifungal resistance.

**METHODS:**

High-resolution genotyping was performed to assess genetic relatedness between isolates. Microbroth dilution was performed on all isolates and underlying mechanisms resistance were inspected with whole genome sequencing.

**RESULTS:**

Eight cases from seven different hospitals were reported, mainly collected from non-sterile sites, in addition to the first documented prosthetic joint infection by *C. auris*. Only two patients reported travel history. Three clades were found, with the first report of Clade IV in Europe. For one patient, initial isolates were pan-susceptible but after short exposure to fluconazole became resistant with a novel mechanism.

**CONCLUSION:**

*C. auris* reported in Czechia in patients without travel history suggests autochthonous spread. Three clades were present, often with unknown route of introduction. Development of fluconazole resistance upon brief exposure highlights the ability of *C. auris* to rapidly evolve.

**Key public health message**

**What did you want to address in this study and why?**

*Candidozyma auris* has rapidly emerged as a major fungal pathogen, displaying high levels of antifungal resistance and frequently causing outbreaks. In this study we report the current situation of *C. auris* in Czechia to inform healthcare agencies and aid local guidelines.

**What have we learnt from this study?**

Cases of *C. auris* are spreading locally in Czechia as most patients did not report any travel history. Molecular genotyping showed the presence of three clades, with Clade IV found for the first time in Europe. Likely, a novel mechanism for fluconazole resistance was found, possibly involving the deletion of the sterol carrier protein 2 gene. One invasive case was noted (infection of a prosthetic joint), the first report of this type of infection.

**What are the implications of your findings for public health?**

As *C. auris* clearly established itself within Czechia and is spreading, healthcare settings need to be aware of this pathogen. Infection prevention policies should be tailored to avoid future outbreaks and hospitals should screen patients to prevent invasive infections. The novel resistance mechanism emphasises the ability of *C. auris* to acquire resistance in diverse ways, stressing the need for optimal diagnostic and antifungal stewardship.

## Introduction

The yeast *Candidozyma auris* (formerly *Candida auris*) has rapidly emerged as an important causative agent of invasive infections within healthcare settings [1]. Due to its ability to colonise human skin and its prolonged survival in the hospital environment and medical devices, this pathogen is capable of causing persistent clonal outbreaks [1]. Moreover, high resistance rates to azoles and an increasing incidence of multidrug resistance limits treatment options and further complicates patient management [2]. Antifungal resistance is often due to point mutations in drug targets, such as *ERG11* for azoles and *FKS1* for echinocandins [2]. Additionally, other mechanisms, including increased efflux pump activity and cell membrane alterations, have been reported [3]. Given these characteristics, the World Health Organization (WHO) listed *C. auris* as a critical fungal pathogen, stressing the need for continuous surveillance, adequate infection prevention measurements and extensive research [4]. To date, six genetically distinct clades have been defined by whole genome sequencing (WGS), with differences in resistance rates, outbreak causing capacity and clinical manifestation [5]. Clades I (South Asia), III (Africa) and IV (South America) are most clinically relevant, given global prevalence, frequent occurrence of outbreaks and high antifungal resistance rates [6].

In Europe, the first case of *C. auris* candidaemia was retrospectively identified in 2007 in France [7] and the first reported outbreak took place in the United Kingdom (UK) between 2015 and 2016 [8]. Since then, this yeast has been reported in at least 19 European countries, with the epidemiological situation in some countries even classified as endemic [9]. While infections are more common in Southern Europe [10], the observed low prevalence in Central and Eastern Europe could be due to limited diagnostic capabilities and difficulties in species determination of yeasts [11]. Biochemical and phenotypic methods may prove inadequate for identifying *C. auris*. Currently, matrix assisted laser desorption-ionisation time-of-flight mass spectrometry (MALDI-TOF MS), chromagar or internal transcribed spacer (ITS) sequencing are needed [1]. While *C. auris* has been rarely found in Central European countries, a few cases have been reported, including a single colonisation case in Czechia in 2019 [9]. While *C. auris* screening and active surveillance at national level is voluntary, the Czech Mycology Reference Laboratory for Antifungals requests that hospital laboratories send samples of all *C. auris* isolates from bloodstream infections and other proven invasive candidiasis cases and strains with acquired antifungal resistance. Isolates from colonised patients can also be submitted. Given that reporting is not mandatory, the number of both invasive and colonised patients may be under-reported.

Here, we describe the rise of *C. auris* in Czechia and characterise the current clinical and genetic epidemiology, in addition to resistance investigation.

## Methods

### Isolates and patient information

All *C. auris* isolates submitted to the Czech Mycology Reference Laboratory for Antifungals between August 2022 and December 2024 were identified by MALDI-TOF MS as previously described and the isolates were stored at -70 °C according to standard procedures [12]. Patient information, including anatomical location, comorbidities, antimicrobial treatment and travel history were retrieved from the hospital electronic patient files linked to each case.

### Short tandem repeat genotyping and whole genome sequencing

Isolates were cultured at the Radboudumc-CWZ (Radboud University Medical Center / Canisius-Wilhelmina Hospital) Center of Expertise for Mycology in the Netherlands at 35 °C on Sabouraud dextrose agar (SDA) plates (Oxoid, Basingstoke, UK) for 2 days. For short tandem repeat (STR) genotyping, DNA was extracted using the MightyPrep Reagent (Takara Bio Inc., Shiga, Japan) according to manufacturer’s instructions. Next, multiplex PCR was performed using 1x Terra PCR Direct Buffer, forward and reverse primers (0.1 – 1 µ), 1.25 U Terra PCR Direct Polymerase Mix (both Takara Bio Inc.), sterile water and isolated DNA. PCR products were run on a 3500 XL genetic analyser (Applied Biosystems, Foster City, United States (US)) and copy numbers were called using GeneMapper 5 software (Applied Biosystems). Genetic relatedness between isolates was determined and visualised with BioNumerics software version 7.6.1 (Applied Maths, Sint-Martems-Latem, Belgium) as previously described [13]. For WGS, DNA was extracted on a MagNA Pure 96 instrument (Roche Diagnostics, Mannheim, Germany) using the Small Volume protocol. Next, genomic libraries were prepared and sequenced with the Illumina MiniSeq platform (Illumina, San Diego, US) with 2-by-150 bp paired-end read mode at Radboud University Medical Center (Radboudumc, Nijmegen, The Netherlands). Read data were aligned with the *C. auris* reference genome B11220 (GCA_003013715.2) using BWA-MEM version 0.7.17 [14] and binary alignment map (BAM) files were filtered as described previously [15]. Single nucleotide polymorphisms (SNPs) were called using Freebayes version 1.3.6 and visualised with a validated variant calling pipeline [15]. Read alignment data were visualised and SNPs in subtelomeric regions (10,000 bp at the end of chromosomes) were excluded in the phylogenetic tree. For all six clades, three isolates per clade were included as controls, with accession numbers listed in Supplementary Table S1.

### Antifungal susceptibility testing and gene inspection

In vitro antifungal susceptibility testing (AFST) against eight antifungals was performed following the European Committee on Antimicrobial Susceptibility Testing (EUCAST) Eukaryotic definitive broth microdilution test method (E.def) version 7.4 [16]. Microtitre plates were incubated at 35 °C for 24 hours and minimum inhibitory concentrations (MICs) were read with a spectrometer at 530 nm. Isolates were classified as wild type or non-wild type according to proposed epidemiological cut-off values (ECOFF) [17]. Additionally, AFST was performed according to Clinical Laboratory Standards Institute (CLSI) M27-S4 guidelines and interpretive categories put forth by the US Centers for Disease Control and Prevention (CDC) were implemented [18]. As quality control strains, *Pichia kudriavzevii* ATCC 6258 and *Candida parapsilosis* ATCC 22019 were included. Hotspot areas in *ERG11* and *FKS1* were amplified with primers as described previously [19]. Amplicons were purified with the AmpliClean and D-Pure protocols (NimaGen, Nijmegen, the Netherlands) and subsequently sequenced on a 3500 XL genetic analyser (Applied Biosystems). With WGS alignments, resistance-associated genes *ERG11* (OL742093.1), *FKS1* (OQ632644.1), *TAC1b* (OL742107.1), *UPC2* (B9J08_000270), *ERG3* (OK564587.1) and *MRR1* (OM287108.1) were located in the *C. auris* B11220 reference genome via nucleotide (nt) Basic Local Alignment Search Tool (BLAST) (https://blast.ncbi.nlm.nih.gov/Blast.cgi). Next, isolates were visually inspected for missense mutations in these genes and other regions via integrative genomics viewer (IGV) version 2.19.1 [20]. Copy number variation was assessed using the Yeast Mapping Analysis Pipeline (YMAP) [21].

### Mutation rate experiments

Fluctuation tests were performed by inoculating 30 independent 200 µL cultures in a StarLab (Hamburg, Germany) CytoOne 96-well plate from a single colony grown for 2 days on Sabouraud dextrose agar (Formedium, Swaffham, UK). Cultures were grown for 24 hours at 120 rpm at 37 °C. Independent cultures were plated onto Sabouraud dextrose agar with 100 µM 5-flucytosine (Fluorochem, Hadfield, UK) on a 6-well plate (StarLab). Total cell count in three independent wells were plated onto Sabouraud dextrose agar to assess total cell density. The 6-well plates were incubated at 37 °C for 2 days. Resistant colonies for individual wells were counted. An estimate of the number of mutation events was calculated from the distribution of the number of mutants using the flan R package [22].

## Results

### Clinical characteristics

**Figure 1:**
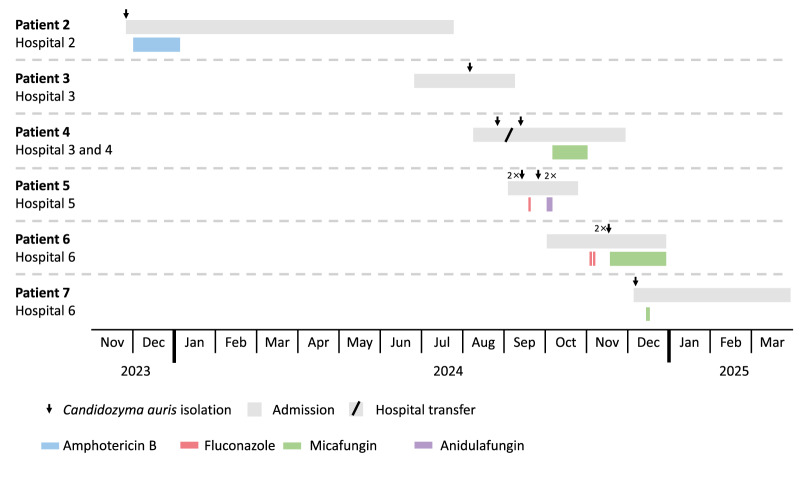
Timeline of admitted patients with *Candidozyma auris* including admission and antifungal therapy, Czechia, August 2022–December 2024

Between August 2022 and December 2024, *C. auris* was found in a total of eight patients from seven different hospitals in Czechia, mostly in the capital, Prague. A total of 13 isolates were sent to the Public Health Institute in Ústí nad Labem for further investigation. All patients were older than 50 years of age and were undergoing treatment for various medical reasons. Twelve isolates were cultured from non-sterile sites during routine testing, while for one patient (Patient 6), *C. auris* was found in a total hip endoprosthesis following a joint puncture (Table 1). Some patients were treated with antifungals, mostly after the isolation of *C. auris*, i.e. liposomal amphotericin B in Patient 2, fluconazole in Patients 5 (also anidulafungin) and 6, and micafungin in Patients 4, 6 (prior to isolating *C. auris*) and 7 (Figure 1). For five patients, *C. auris* was isolated several weeks after hospital admission. All eight patients had prior antibiotic exposure and only Patient 8 was not admitted. Patients 2 and 4 had been previously admitted to a hospital in a southern African and southern European country, respectively. The only patient with a *C. auris* infection (Patient 6), which was found in a prosthetic hip joint, was admitted to the orthopaedic and intensive care unit (ICU). This patient was treated with 100 mg micafungin every day for 35 days and on day 20, surgical exploration of the hip prosthesis was performed.

**Table 1:** Clinical overview of *Candidozyma auris* cases, Czechia, August 2022–December 2024 (n = 8, 1 female, 7 male)

Patient	Hospital	Isolate ID	Isolation date (days after admission)	Source	Travel history	Clade
1	1	C39/22	Aug 2022 (day 17)	Urinary catheter	None	III
2	2	C654/24	Nov 2023 (day 1)	Urine	Southern Africa (hospitalisation)	III
3	3	C864/24	Aug 2024 (day 44)	Oral cavity	None	IV
4	3	C909/24	Aug 2024 (day 16)	Urinary catheter	Southern Europe (repatriated)	I
	4	C958/24	Sep 2024 (day 30)	Urine		I
5	5	C985/24	Sep 2024 (day 1)	Urinary catheter	None	IV
		C986/24	Sep 2024 (day 2)	Rectum		IV
		C1083/24	Oct 2024 (day 22)	Urinary catheter		IV
		C1084/24	Oct 2024 (day 24)	Urinary catheter		IV
6	6	C1230/24	Nov 2024 (day 1)	Subcutis puncture	None	I
		C1231/24	Nov 2024 (day 1)	Total hip endoprosthesis Synovial fluid aspiration	None	I
7	6	C1321/24	Dec 2024 (day 27)	Urine	None	I
8	7	C1167/24	Nov 2024 (day 1)	BAL	None	IV

BAL: bronchoalveolar lavage; ID: identification.

### Molecular genotyping with short tandem repeat analysis

**Figure 2:**
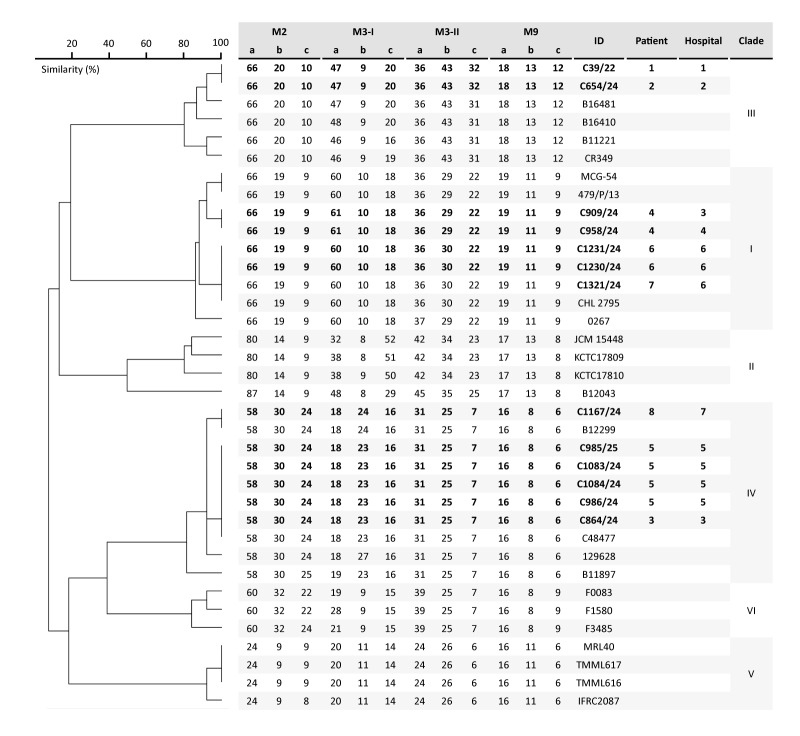
Unweighted pair group method with arithmetic means dendrogram based on short tandem repeat genotyping of 13 *Candidozyma auris* isolates, with control isolates from six known clades, Czechia, August 2022–December 2024

To investigate genetic relatedness, STR genotyping was performed on all 13 isolates. This showed five different genotypes and the presence of three clades (I, III and IV) in Czechia (Table 1, Figure 2). Isolates from Patients 1 and 2 were designated to Clade III and demonstrated identical genotypes. Isolates from Patients 3, 5 and 8 were all found to be Clade IV, with Patient 3 and Patient 5 having identical genotypes. The genotype of the isolate from Patient 8 differed from Patient 3 and Patient 5 by only one marker with one copy number. Isolates from Patients 4, 6 and 7 were allocated to Clade I and had different genotypes, with the two isolates from Patient 4 differing by one copy number in two STR markers with the isolates of Patient 6 and Patient 7.

Branch lengths indicate phylogenetic relatedness according to short tandem repeat markers.

Isolates genotyped in the current study are indicated in bold.

### Antifungal resistance and mechanisms

In vitro AFST was performed according to EUCAST guidelines against eight antifungals and resistance-associated genes were investigated for nonsynonymous mutations (Table 2). Of 13 isolates, 10 isolates displayed a MIC of ≥ 64 mg/L against fluconazole, and three isolates (all Clade IV) had a MIC of 4–8 mg/L against fluconazole. High fluconazole MICs coincided with *ERG11^Y132^* and *ERG11^VF125AL^* (also referred to as F126L) in Clades I and III, respectively. For the isolates with high fluconazole MICs in Clade IV, no mutations were found in *ERG11.* Interestingly, four Clade IV isolates were collected from Patient 5, with the first two isolates showing low fluconazole MICs, while the following two isolates had an MIC of 64 mg/L. The isolation of the latter two isolates occurred after two doses of fluconazole (200mg), administered the day before surgery. When applying proposed ECVs, all isolates were classified as wild type against amphotericin B. Two isolates originating from the same patient (Patient 4) who was previously exposed to micafungin were non-wild type for echinocandins and harboured *FKS1^S639P^* mutations. The other isolates were classified as echinocandin wild type. Additionally, AFST according to CLSI guidelines yielded comparable results, with raw MICs shown in Supplementary Table S2.

**Table 2:** Antifungal minimum inhibitory concentrations of 13 *Candidozyma auris* isolates according to European Committee on Antimicrobial Susceptibility Testing guidelines^a^, Czechia, August 2022–December 2024

			Antifungal minimum inhibitory concentrations (mg/L)									
Patient	Isolate ID	Clade	AMB	FLU	ITC	VOR	POS	ISA	AFG	MFG	*ERG11*	*FKS1*
1	C39/22	III	0.5	64	0.125	1	0.03	0.06	0.06	0.03	VF125AL	WT
2	C654/24	III	0.5	64	0.125	0.25	0.03	0.03	0.06	0.06	VF125AL	WT
3	C864/24	IV	1	8	0.06	0.06	0.03	0.03	0.06	0.06	WT	WT
4	C909/24	I	1	≥ 64	0.125	1	0.06	0.06	2	1	Y132F	S639P
4	C958/24	I	0.5	≥ 64	0.25	1	0.03	0.06	2	2	Y132F	S639P
5	C985/24	IV	1	8	0.06	0.03	0.016	0.03	0.03	0.03	WT	WT
5	C986/24	IV	1	4	0.06	0.03	0.03	0.03	0.03	0.06	WT	WT
5	C1083/24	IV	1	64	0.25	0.25	0.125	0.25	0.06	0.06	WT	WT
5	C1084/24	IV	1	64	0.125	0.25	0.125	0.125	0.06	0.06	WT	WT
6	C1230/24	I	1	≥ 64	0.25	0.5	0.06	0.125	0.06	0.06	Y132F	WT
6	C1231/24	I	1	≥ 64	0.25	1	0.06	0.125	0.03	0.06	Y132F	WT
7	C1321/24	I	0.5	≥ 64	0.25	0.5	0.125	0.125	0.06	0.03	Y132F	WT
8	C1167/24	IV	1	64	0.25	0.5	0.25	0.5	0.06	0.06	WT	WT
**Range antifungals**			0.5–1	4–≥ 64	0.06–0.25	0.03–1	0.016–0.25	0.03–0.5	0.03–2	0.03–2	NA	NA

AMB: amphotericin B; AFG: anidulafungin; FLU: fluconazole; ID: identification; ISA: isavuconazole; ITC: itraconazole; MFG: micafungin; NA: not applicable; POS: posaconazole; VOR: voriconazole; WT: wild type.

^a^ European Committee on Antimicrobial Susceptibility Testing (EUCAST) Eukaryotic definitive broth microdilution test method (E.DEF) version 7.4.

Clades were determined by short tandem repeat genotyping.

### Whole genome sequencing single nucleotide polymorphism analysis

**Figure 3:**
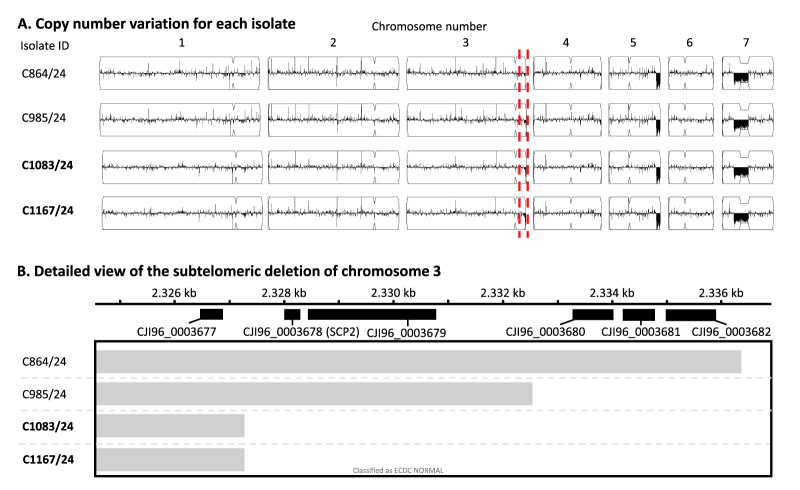
Copy number variation analysis of *Candidozyma auris* isolates, Czechia, August 2022–December 2024 (n = 4)

**Figure 4:**
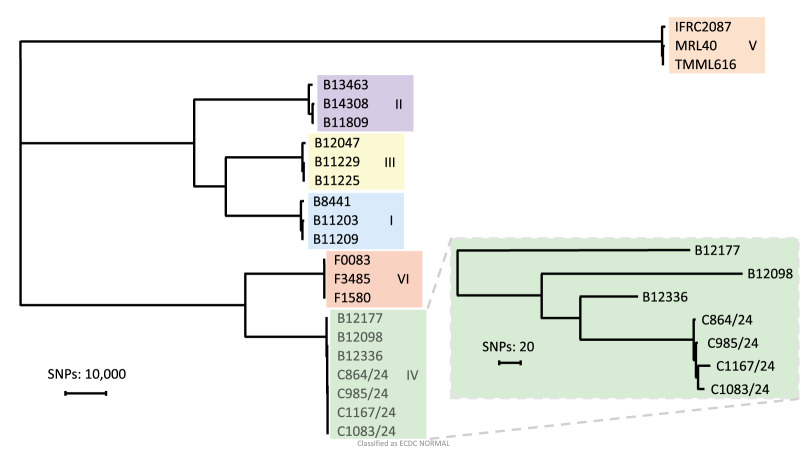
Phylogenetic tree based on whole genome sequencing single nucleotide polymorphisms of 22 *Candidozyma auris* isolates spanning all six described clades, Czechia, August 2022–December 2024

To identify the molecular mechanism causing fluconazole resistance in Clade IV isolates and to determine whether these Clade IV isolates, reported in Europe for the first time, were due to single or multiple introductions in Czechia, WGS SNP analysis was performed. The analysis included a selection of four Clade IV isolates, spanning three patients (Patients 3, 5 and 8) from three different hospitals (Hospitals 3, 5 and 7). From Patient 5, the first susceptible and resistant isolate was included. Surprisingly, all azole resistance-associated genes *ERG11*, *TAC1b*, *MRR1*, *ERG3* and *UPC2* harboured no nonsynonymous mutations in susceptible and resistant isolates. Additionally, no copy number variation was observed for all investigated isolates with the exception of subtelomeric deletions at the end of chromosome 3, which were found in three fluconazole-resistant isolates, but not in the susceptible isolate C864/24 (Figure 2). In the fluconazole-susceptible isolate C985/24, a subtelomeric deletion of three genes was found, while in the fluconazole-resistant isolates C1083/24 and C1167/24, there was an extended deletion comprising two additional genes CJI96_0003678 and CJI96_0003679. By excluding the subtelomeric deletion region from the subsequent SNP analysis, all Czech isolates were shown to form a monophyletic branch (Figure 3). Single nt polymorphism differences between isolates, all collected within 96 days, ranged from 7 to 15 SNPs (Figure 4). The two most closely related isolates (C864/24 and C985/24) differed by 3 SNPs and were collected 34 days apart from patients admitted to Hospital 3 and 5, both located in Prague. The fluconazole resistant isolate C1083/24 differed by only 7 SNPs from the susceptible isolate C985/24, which was collected 21 days earlier in the same patient (Patient 5). The other resistant isolate C1167/24, from Patient 8, differed by 15 SNPs from isolate C1083/24, both harbouring the same subtelomeric deletion, and was collected 41 days later in another hospital in Prague. Notably, isolate C1167/24 differed by only 10 SNPs from isolate C985/24, collected 62 days earlier from Patient 5.

ID: identification.

Isolates indicated in bold are fluconazole resistant.

Red dotted lines indicate the area of Panel A that is shown in detail in Panel B.

**Figure 5:**
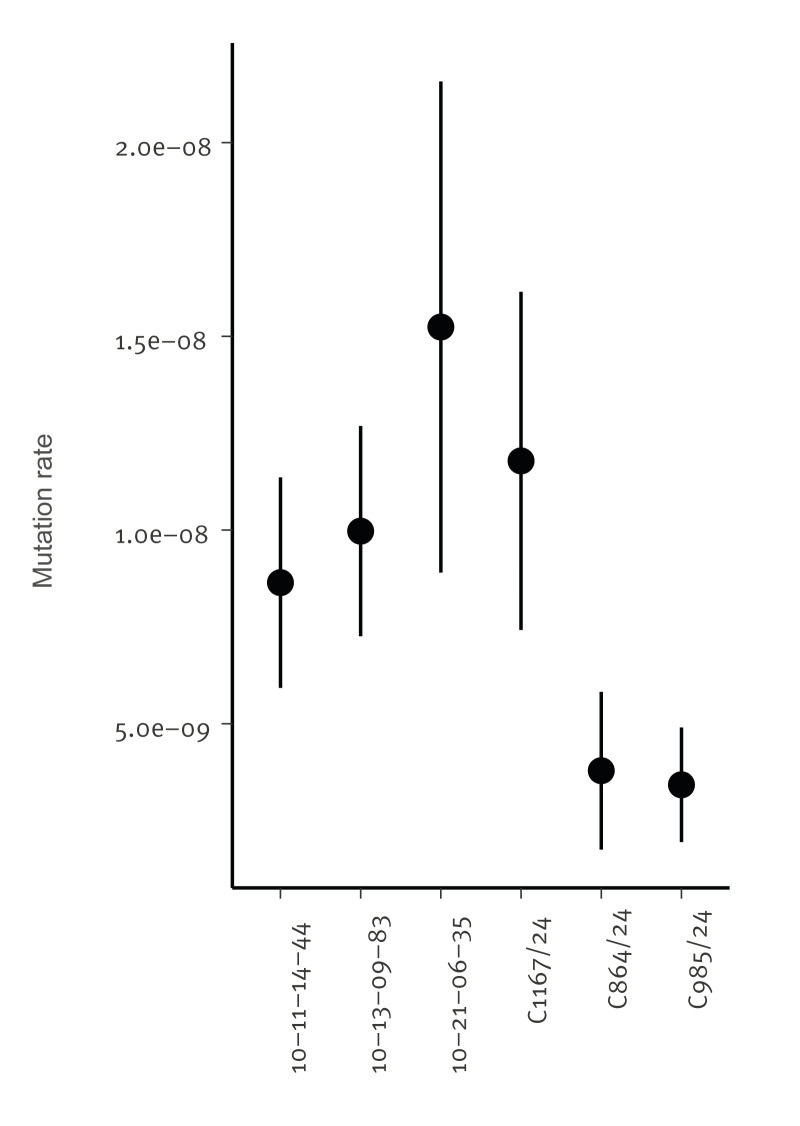
Mean mutation rate of *Candidozyma auris* isolates and Clade IV control isolates, Czechia, August 2022–December 2024 (n = 20)

A mutation rate analysis of three of the outbreak isolates (C864/24, C985/24 and C1167/24) and three unrelated Clade IV isolates revealed that the three outbreak isolates differed in their mutation rate (Figure 5).

The fluconazole susceptible isolates C864/24 and C985/24 showed a mutation rate of 3.8 x 10 ^− 9^ and 3.4 x 10 ^− 9^ per cell division, respectively, while the fluconazole resistant isolate C1167/24 had an elevated mutation rate of 1.2 x 10 ^− 8^ per cell division. The latter mutation rate was comparable to the other Clade IV isolates.

Mutation rate analysis from fluctuation tests of *C. auris* isolates. Mean mutation rate with 95% confidence intervals are shown.

## Discussion

The first *C. auris* case in Czechia was reported in 2019 and reflected colonisation [9]. For seven of the eight cases in our study, *C. auris* was isolated from non-sterile sites. Yeasts, including *C. auris*, are able to colonise humans and antifungal treatment is not needed [23,24]. Nonetheless, patients colonised with *C. auris* pose a possible cross-transmission risk within healthcare settings and should be nursed in isolation to minimise the risk of spreading [25]. Two patients (Patient 2 and Patient 4) received medical treatment abroad, in southern Africa and southern Europe, respectively. These areas are known to have a high prevalence of *C. auris* within healthcare settings [9,10], therefore these patients likely imported the pathogen. The other six patients had no history of travel, suggesting circulation of *C. auris* within the country, especially within Prague. In addition to the colonised cases, the first deep wound infection involved a prosthetic joint. The most common manifestation of invasive yeast infections involves the bloodstream, while joints like the knee or hip are rarely infected by yeasts [26]. To the best of our knowledge, this is the first patient with *C. auris* as the infectious aetiological agent described in Europe [26]. The patient was initially treated with micafungin in combination with the removal of the hip prosthesis. Echinocandins have better biofilm activity and safety profile than azoles, although data on treatment effectiveness of echinocandins in such infections are limited. *Candida* prosthetic joint infections require a prolonged treatment time and often have poor outcome, especially if an implant cannot be removed [26]. Here, the patient relapsed 3 months later, after which they underwent surgical debridement of the femoral head and were treated with 100 mg micafungin daily for 3–6 months.

Czechia has a National Reference Centre for Healthcare Associated Infections (NRC HAI) and, since 2022, two National Reference Laboratories for medical mycology that monitor epidemiology in the country. The NRC HAI prepares guidance documents and educational support for hospital staff in the field of prevention of hospital acquired infections. This includes infection prevention and control measures and antimicrobial stewardship to detect and manage nosocomial infections including *C. auris*. These three reference institutions have actively collaborated on *C. auris* since the cases described here were detected in 2022. The hospitals involved were informed and seminars for microbiologists about a worsening situation were prepared. Local guidance for the management of *C. auris* in healthcare settings is has been published recently, including the use of chromogenic media for *C. auris* detection and screening selection criteria, with emphasis on infection prevention measures in hospitals [27].

Microbiologists were advised to support local medical staff with relevant information and recommendations regarding *C. auris* outbreak prevention and patient management. In a situation when there is not enough personnel or structural support to isolate a *C. auris*-positive patient, as in most healthcare facilities, it is recommended to adapt the already applied measures against common hospital infections. For *C. auris*, contact isolation, measures comparable to meticillin-resistant *Staphylococcus aureus* (MRSA) are recommended, with emphasis on specific environmental disinfection conditions using sporicidal disinfectants. Local outbreak management is coordinated by local staff and is not uniform for all hospitals. The application of proper infection control measures is strongly influenced by the local organisation of healthcare in the facility and the availability or shortage of single rooms and staff. Mandatory notification of cases to national ministries of health or national outbreak policies are warranted as the lack of these likely complicates containment of this pathogen.

As reference laboratories in Czechia do not yet have established methods for prospective molecular genotyping or WGS on *C. auris*, all isolates were retrospectively investigated with STR genotyping first, followed by WGS on a selection of isolates. With STR genotyping, we showed the presence of three clades within our country. To date, European countries have only reported Clades I and III [10,28], making this study the first to report Clade IV in Europe. While Clade IV isolates are found more frequently in the Americas, reports on other continents remain limited [28-30].

The low number of SNPs, especially the 3 SNP difference seen within 34 days between isolates C864/24 and C985/24 from patients in Hospital 3 and Hospital 5 suggests transmission between hospitals [28]. The 10 and 15 SNP difference between the isolates of patient 5 (C985/24 and C1083/24, respectively) and C1167/24 from Hospital 5 and Hospital 7 were around the cutoff value described earlier for *C. auris* [31]. It is therefore not evident whether there was clonal transmission between these patients or whether they originated from another common source. Of note, some personnel are employed in multiple hospitals which could potentially act as a transmission route, although this was neither evaluated nor confirmed for the current case. Another transmission event could have taken place between the two Clade III patients but since the first patient from 2022 reported no travel history and the second patient reported a direct link a year later to a country in southern Africa, being endemic for Clade III [29], both cases are likely epidemiologically unrelated. However, WGS SNP analysis would be needed to confirm this [32], and to also confirm the likely clonal transmission of Clade I isolates from Patient 6 and Patient 7, who were nursed in the same department and harboured identical STR genotypes.

Fluconazole resistance was present in all Clade I and III isolates with the commonly reported *ERG11* mutations Y132F and VF125AL that are known to confer resistance [2]. Additionally, elevated echinocandin MICs were found in one isolate harbouring the common *FKS1^S639P^* mutation [31]. From the six Clade IV isolates, three were found to be susceptible to fluconazole, which is in agreement with other studies reporting lower resistance rates for Clade IV isolates as compared with Clades I and III [6]. Interestingly, initial isolates from Patient 5 were susceptible, while 21 days later resistant isolates were found after the patient had been treated with two doses of fluconazole within 24 hours. This fluconazole resistance was not caused by known resistance mechanisms, but seemed to involve a 5,000-bp subtelomeric deletion at the end of chromosome 3. This region contains two genes, of which one is *SCP2* and involved in cellular physiology via the binding of lipids, including ergosterol, the target of azoles [33]. Although it should be investigated whether the deletion of this gene caused fluconazole resistance, mechanistically *SCP2* is a plausible candidate. Besides the subtelomeric deletion, WGS SNP analysis demonstrated only a 7 SNP difference between the susceptible and fluconazole resistant isolate of Patient 5, which seems to make short-term, therapy-induced resistance a likely explanation. However, fluconazole resistance development within patients is rarely reported and when documented often involves prolonged exposure [34]. Moreover, if the subtelomeric deletion tentatively causing fluconazole resistance developed in this patient, then clonal transmission between Patient 5 and Patient 8 is likely, as isolate C1167/24 of Patient 8 had an identical subtelomeric deletion on chromosome 3. The difference of 15 SNPs and collection days within 2 months of each other is rather high and usually requires more time to accrue [32]. However, the elevated mutation rate of isolate C1167/24 compared with earlier susceptible isolates would suggest that this elevated mutation rate led to the rapid accruement of SNPs or indels observed here and point towards a mutator phenotype of this particular isolate, as has been observed in other fungal species [35-37]. This indicates the resistance was acquired during the short-term therapy, thereby also gaining an elevated mutation rate that is responsible for the high number of SNPs.

The main limitation of this study was the lack of epidemiological investigation, including environmental sampling. As *C. auris* is known to colonise hospital environments [8], such investigation might have uncovered potential transmission routes and allow adequate infection-prevention measures to be taken.

## Conclusion

*Candidozyma auris* is spreading within Czechia. While most detections described in this report involved colonised patients, we also identified a deep prosthetic joint infection. Three genomic clades are present within the country, with the first known occurrence of Clade IV in Europe. Fluconazole resistance development with a novel mechanism raises concerns about the already increasing resistance rate for *C. auris*.

## Data Availability

Raw read data generated in the current study were submitted to the National Center for Biotechnology Information (NCBI) Sequence Read Archive (SRA) database under BioProject ID PRJNA1217383. All nt sequences generated in the current study were deposited in the NCBI GenBank (accession numbers PV037699-PV037716).

## Supplementary Data

90000 Supplementary Material
