# Supplementary material for: The rise of Candidozyma auris in Czechia: three clades, prosthetic joint infection and fluconazole resistance development, 2022 to 2024
Source: Euro Surveill. 2025 Nov 13;30(45):2500285. doi: 10.2807/1560-7917.ES.2025.30.45.2500285 (PMC12633708; doi:10.2807/1560-7917.ES.2025.30.45.2500285)
Supplement: Supplementary Material [file 25-00285_MEIJER_Supplement.pdf]

This supplementary material is hosted by *Eurosurveillance* as supporting information alongside the article ‘The rise of *Candidozyma auris* in Czechia: three clades, prosthetic joint infection and fluconazole resistance development, 2022 to 2024’, on behalf of the authors, who remain responsible for the accuracy and appropriateness of the content. The same standards for ethics, copyright, attributions and permissions as for the article apply. Supplements are not edited by *Eurosurveillance* and the journal is not responsible for the maintenance of any links or email addresses provided therein.

Supplementary Table S1. Overview of *Candida auris* control isolates included in the whole genome sequencing (WGS) analysis.

ID	SRA	Country	Clade
B8441	SRR10851769	Pakistan	I
B11203	SRR14252434	India	I
B11209	SRR3883441	India	I
B11809	SRR10461262	South Korea	II
B13463	SRR10461159	Canada	II
B14308	SRR10461147	USA	II
B11225	SRR3883457	South Africa	III
B11229	SRR3883457	South Africa	III
B12037	SRR10461253	Canada	III
B12098	SRR10461248	Panama	IV
B12177	SRR10461201	Venezuela	IV
B12336	SRR7140028	Colombia	IV
IFRC2087	SRR9007776	Iran	V
MRL40	SRR18325430	Iran	V
TMML616	SRR18325431	Iran	V
F0083	SRR25455197	Singapore	VI
F1580	SRR25455198	Singapore	VI
F3485	SRR25455199	Singapore	VI

Supplementary Table S2. Antifungal minimum inhibitory concentrations (MICs) of 13 *Candida auris* isolates according to CLSI M27-S4 guidelines. MICs are displayed in μg/mL and the clade was determined according to short tandem repeat genotyping.

Pt #	ID	Clade	AMB	FLU	ITC	VOR	POS	ISA	AFG	MFG	*ERG11*	*FKS1*
1	C39/22	III	0.25	32	0.063	1	≤0.016	≤0.016	0.031	0.031	VF125AL	WT
2	C654/24	III	1	32	0.063	0.25	≤0.016	0.031	0.063	0.063	VF125AL	WT
3	C864/24	IV	1	4	0.063	0.031	≤0.016	0.031	0.063	0.063	WT	WT
4	C909/24	I	1	64	0.063	0.5	≤0.016	0.031	1	1	Y132F	S639P
4	C958/24	I	1	32	0.063	0.5	≤0.016	0.031	2	1	Y132F	S639P
5	C985/24	IV	1	4	0.063	≤0.016	≤0.016	≤0.016	0.016	0.031	WT	WT
5	C986/24	IV	1	4	0.063	≤0.016	≤0.016	≤0.016	0.016	0.031	WT	WT
5	C1083/24	IV	1	32	0.25	0.25	0.063	0.125	0.063	0.063	WT	WT
5	C1084/24	IV	1	32	0.125	0.25	0.063	0.125	0.063	0.063	WT	WT
6	C1230/24	I	1	64	0.125	0.5	0.031	0.063	0.063	0.063	Y132F	WT
6	C1231/24	I	0.5	64	0.125	0.5	0.031	0.063	0.031	0.063	Y132F	WT
7	C1321/24	I	0.5	64	0.25	0.5	0.125	0.125	0.063	0.031	Y132F	WT
8	C1167/24	IV	1	32	0.25	0.5	0.125	0.5	0.063	0.063	WT	WT
